# Simple particle shapes for DEM simulations of railway ballast: influence of shape descriptors on packing behaviour

**DOI:** 10.1007/s10035-020-1009-0

**Published:** 2020-03-23

**Authors:** Bettina Suhr, Klaus Six

**Affiliations:** Virtual Vehicle Research GmbH, Inffeldgasse 21/A, Graz, 8010 Austria

**Keywords:** DEM modelling, Particle shape, Efficiency, Railway ballast, Packing behaviour

## Abstract

**Abstract:**

In any DEM simulation, the chosen particle shape will greatly influence the simulated material behaviour. For a specific material, e.g. railway ballast, it remains an open question how to model the particle shape, such that DEM simulations are computationally efficient and simulation results are in good accordance with measurements. While DEM shape modelling for railway ballast is well addressed in the literature, approaches mainly aim at approximating the stones’ actual shape, resulting in rather complex and thus inefficient particle shapes. In contrast, very simple DEM shapes will be constructed, clumps of three spheres, which aim to approximate shape descriptors of the considered ballast material. In DEM simulations of the packing behaviour, a set of clump shapes is identified, which can pack at porosities observed at track sites, as well as in lab tests. The relation between particle shape (descriptors) and obtained packing (characteristic) is investigated in a correlation analysis. The simulated packing’s porosity is strongly correlated to four shape descriptors, which are also strongly correlated among each other. Thus, to derive simple shape models of a given particle shape, matching one of these shape descriptors, might be a good first step to bring simulated porosities closer to measured ones. The conducted correlation analysis also shows that packing’s coordination number and isotropic fabric are correlated to more shape descriptors, making it more difficult to estimate the effect of particle shape on these quantities.

**Graphic abstract:**

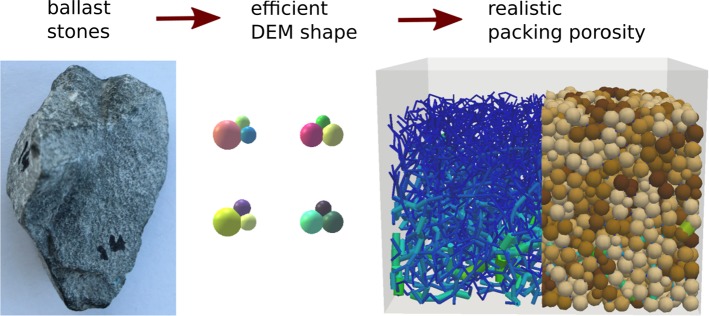

**Electronic supplementary material:**

The online version of this article (10.1007/s10035-020-1009-0) contains supplementary material, which is available to authorized users.

## Introduction

In Discrete Element Method (DEM) simulations, the choice of particle shape and size, the applied contact model with its material parameters and the packing preparation method will determine the simulated bulk behaviour of the material. When a specific material is considered, it is a challenge to separate the influence of particle shape and material parameters. In this work, shape modelling for railway ballast will be addressed, while the parametrisation of the contact model is subject of future work.

The shape of two types of railway ballast, Calcite and Kieselkalk, was analysed in [51]. The stones under investigation were composed of sharp corners and edges as well as rounded areas and their angularity was analysed. Another important shape characteristic of ballast stones is their non-convexity, which was evaluated in [51] using the convexity index. Both sharp corners and edges as well as non-convex shapes can be expected to significantly influence the ballast bulk behaviour as they yield a big interlocking potential.

*Particle shape analysis* in general is usually conducted on three different scales: form, angularity/roundness and texture. So called overall shape parameters correlate to more than one of the above scales. Particle form is described by 1D form factors, which are calculated involving the particle’s longest, intermediate and shortest axes (*L*, *I*, and *S* respectively), see [4] for an overview of form factors. Widely used factors include elongation $e=I/L$ and flatness $f=S/I$ and differently defined aspect ratios. Many of the other form factors correlate with elongation or flatness, see [3]. Roundness was firstly defined by Wadell, [57–59], but also other definitions exist, see [4] for 2D data or [5, 32, 33, 62, 64] for 3D data. For roundness computation only convex areas of the particle are considered, ignoring edges and corners in concave parts. In contrast, the concept of angularity considers all corners and edges of a particle. Angularity is frequently investigated using 2D data, [2, 30, 36, 60], but also different approaches using 3D data are available in the literature, e.g. [13, 26, 30, 61]. Texture is traditionally analysed from black/white or gray-scale pictures, i.e. 2D data, see [2] for a comparison of different approaches. In [61], a 3D surface texture index for 3D voxel meshes was developed. Overall shape parameters are sphericity and convexity, see [62], but also ellipseness, [25], or ellipsoidness, [53] as they can be influenced by form, roundness/ angularity and texture. In general, the characterisation of particle shape is a challenge. From the above remarks it becomes clear that it is often dependent on the available data on grain shape. Moreover, differing definitions are used in the literature for several shape descriptors, i.e. no clear standards exist.

*Particle shape modelling* in DEM simulations started with spherical particles in [8]. An early work involving non-spherical particles is [38]. According to O’Sullivan [34], rigid clusters of disc or spheres were introduced next, with both non-overlapping and overlapping clusters, compare citations given in [34]. This clustering or clumping of spheres is often called multi-sphere method. Within one clump of spheres, the relative positions of the spheres do not change. Forces and torques arising from contacts with other particles are accumulated relative to the centre of mass of the clump, compare e.g. [27]. When spheres are clustered with overlapping volume, it is important to correct the mass and inertia and several approaches have been suggested in the literature. [14] overlaid the clump of spheres with a grid. If one grid element is overlain by more than one sphere, then a multiple consideration of masses is prevented by considering the grid element to calculate mass and moments of inertia of the clump. In [10, 11], the density of the single spheres building the clump is scaled such that the clump has the correct mass and inertia. In [37], an analytical approach for the correct calculation of mass and inertia was presented. This method is used mainly for clumps consisting of two spheres, while it might be difficult to apply it to clumps composed of many, highly overlapping spheres. The multi-sphere method is used for DEM simulations of many different applications, see e.g. [27]. Several other particles types are used for DEM simulations: ellipsoids, super-quadrics, potential particles or polyhedral particles to name the most popular ones. As this paper will deal with clumps of spheres exclusively, the interested reader is referred to [27, 34] for more details. In general, a more detailed shape modelling in DEM simulations corresponds to a higher computational effort.

*Particle shape descriptor’s influence* on the simulated bulk behaviour is a topic of active research. Observed relations between particle shape and certain aspects of bulk behaviour will most certainly be dependent on the considered application. In a loose flow situation other aspects of particle shape might affect the bulk behaviour than in a highly stressed, dense packing. Two examples of different applications are mentioned here to clarify this point. In [21], the effect of grain roughness on simulated quasi-static triaxial test is investigated and compared to experimental results using Karlsruhe sand. 12 clumps of spheres are constructed, where 10 of them are two-dimensional complex shapes and two are three-dimensional complex shapes. Particle roughness (shape) is characterised by an aspect index and two different convexity indices defined as the ratio between the smallest sphere volume encompassing the cluster and the cluster volume, and the ratio between the smallest convex volume encompassing the cluster and the cluster volume. It simulated triaxial tests, it was seen that sand grain roughness could be modelled using irregular shapes, which may cause an increase of strength and volume changes. Also, observed fluctuations in the kinetic energy increased with grain roughness. In [39], the influence of particle shape on the simulated performance of granular dampers is investigated. For this, 14 complex clumps of spheres are defined, where 12 shapes are two-dimensional complex shapes and two are three-dimensional complex shapes. A set of four shape descriptors is applied to characterise shape: aspect ratio, circularity, convexity and solidity. For their calculation the perimeter and area of the different shapes is used, which is approximated using intersections of spherocylindrical shells. It is stated that the set of shape descriptors is chosen because of the study’s aim to quantify the main geometric characteristics influencing the particles rotational behaviour. In simulations of granular dampers in the absence of gravity a weak forcing led to a gas-like behaviour of the granular system. Here, it was found that damping efficiency is smaller for complex shaped than for spherical particles. Also, the damper’s efficiency depended on the aspect ratio and circularity for rod like particles. Although both works cited above use clumps of spheres, there exist differences in the calculation of shape descriptors, e.g. [21] defines convexity with respect to the particle’s volume, while [39] defines it using perimeters of approximating intersections of spherocylinders. Some works even define shape descriptors, which can only be used for the chosen DEM particle type: [63] defines angularity of the polyhedral particles as ratio of the sphericity to the number of vertices. These examples show again the challenge of particle shape characterisation and that care has to be taken when drawing conclusions from different studies.

*Modelling of railway ballast* is addressed in the literature using different approaches and DEM particle types. Up to now, clumps of spheres offer the only computationally efficient way to model non-convex shapes in DEM. For the consideration of sharp edges or smooth surfaces usually a high number of spheres is needed, including spheres with a small radius, which comes at a high computational effort. In an early approach, [28] constructs clumps controlling their sphericity and angularity (resulting clumps consist of more than 20 spheres). In the literature, some works use 2D or 3D data of the considered material to construct clumps, e.g. [10–12, 17, 47]. The constructed clumps are detailed shape models, which usually results in a high number of spheres (above 10 in [17] and above 50 in [11]). In contrast to this, [6] investigated the usage of geometric clumps (tetrahedral or flaky) consisting of up to eight spheres. In [24], results of simulation with complex particle shapes were compared to those using a simple two ball clump and qualitatively similar results were obtained. In [7], modelling of crushed rock with simple clumps (two spheres) or more complex clumps (up to eight spheres) was investigated. The results of compression test or direct shear test simulations, which were obtained with the simple clumps were comparable to those obtained with complex clumps. When particle breakage or asperity breakage is considered, also clumps of bonded spheres are used to model railway ballast, see e.g. [18, 29, 31]. For the DEM simulation of railway ballast polyhedra are also used in the literature. Up to now, all used polyhedra are strictly convex. While non-convex polyhedra recently start to become available in some DEM codes, contact detection is highly computationally demanding and this choice should be made cautiously. An early work on sharply edged particles can be found in [45]. In the approach used by [16, 40, 54], data from 3d-scanned ballast stones can be used to build polyhedral DEM particles, with respect to certain shape descriptors. In [35], polyhedral shapes for railway ballast are constructed by the application of Proper Orthogonal Decomposition to 3D scanner data. This approach inherently yields a control over the level of detail of the constructed shapes. In [1, 15], potential particles for the simulation of triaxial tests of railway ballast are used. [1] present a method to manually adapt the shape of a potential particle to the shape of a ballast stone.

Independent from the particle type chosen, most citations mentioned above used rather complex shapes to model in high detail the complex shape of railway ballast stones. Exceptions, like [6, 7, 24] who use simple shape models, only obtained qualitative not quantitative agreement between simulations and experiments. The usage of simple particle shapes for quantitative comparison with experiments is a continuation of the authors’ previous works. In [48], two types of ballast, named Calcite and Kieselkalk, were tested in direct shear and compression tests. DEM simulations used a “lucky guess” simple particle shape and were combined with an enhanced contact law, taking into account additional physical effects such as breakage of edges or yielding. The obtained simulation results were in very good accordance with the experimental results. A shape analysis of the same two types of ballast was conducted in [51]. Regarding form, angularity, sphericity, convexity index no difference could be seen between both ballast types.

The focus of this work is laid on the systematic construction of simple clump shapes and the analysis of their packing behaviour. Packing porosity as well as coordination number and isotropic fabric, will strongly influence the simulated bulk behaviour. Aiming at simplicity and efficiency, clumps of three spheres will be constructed using information available from the shape analysis, [51]: elongation, flatness, convexity index and sphericity. For the different clump shapes, the packing behaviour will be addressed with respect to porosity (densest and loosest packing to be generated for a shape) coordination number and isotropic fabric. If a clump shape is used to simulate railway ballast, it should be able to generate packings at field porosity as well as lab porosities. While field porosities are hard to measure, porosity $\phi =0.405$ is reported in the literature, see [41], which state: “similar field densities were reported in a previous study:” [18]. For lab testing, usually lower porosities are reported. In this work, clump shapes are searched which can generate (after a pre-compaction step) samples in the range of the experiments reported in [48]: $0.42 \le \phi \le 0.46$. To investigate the simple clump shapes’ packing behaviour is seen as an important first step. In a planned follow up paper, the authors aim to obtain a fully validated DEM model of railway ballast using simple clump shapes. Available experimental data on uniaxial compression and direct shear tests, [50], will be used for this purpose. A methodology for material parameter calibration should be develop to be able to complete the validation process.

This work is organised as follows: In Sect. 2, the shape descriptors of the railway ballast are summarised before the simple shapes are constructed. A correlation analysis of the shape descriptors completes this section. Details of the constructed DEM simulations are given in Sect. 3. Section 4 starts with a correlation analysis between the obtained packings’ characteristics and the clumps’ shape descriptors. The found correlations are shown to be valuable for the choice of clump shapes, used for modelling the two types of railway ballast. In Sect. 5 conclusions are drawn and an outlook to future work is given.

## Particle shape construction

In this work, railway ballast will be modelled using clumps of few spheres. Clumps of three spheres do not allow free rotations as clumps of two spheres and are computationally very efficient. Obviously, clumps of spheres lack the angularity of real ballast stones but they are non-convex, which is an important property of ballast, and also allows for interlocking. Clumps of spheres can be arranged to obtain particles with higher or lower “surface roughness”, [46]. Roth and Jaeger [43] and Irazabal et al. [19] describe particles with corrugated surfaces or cavities between overlapped spheres with the expression “geometric friction”, which is also known to affect the packing porosity. In this work, the clump construction aims to generate high geometric friction to compensate, to some extent, the lack of angularity, i.e. sharp edges and corners. Moreover, clumps are not constructed to approximate the shape of ballast stones with respect to the volume error, but to approximate shape descriptors, which are known from previous work, [51]. Details on all clumps constructed in this paper can be found in the supplemental material provided with this work.

The simulations conducted correspond to experiments, where railway ballast was packed in a box of a direct shear tester, and due to the size of the test rig, the gradation curve of the stones was cut off at 40 mm, see [48]. Two sieve sizes remained: 31.5 mm: 34% and 40 mm: 100%. This gives a sieve size ratio of 0.79 and a ratio of small particles to large particles of 0.5. In [9], poly- and bi-disperse assemblies were simulated in uniaxial compression tests. In general, it was seen that larger particles see larger contact forces compared to smaller particles. However, one of the cases considered was similar to the sizes above: a bi-disperse sample with ratio small and large particles of 0.8 and a ratio of numbers small to large of 0.66. [9] stated: “For the assembly with a radius ratio of 0.8, both the particles are seen to participate in the force transmission without much variation”. Therefore, it is concluded that the computationally efficient use of a mono-disperse sample is justified for the given sieve size curve.

### Summary of shape analysis

**Fig. 1:**
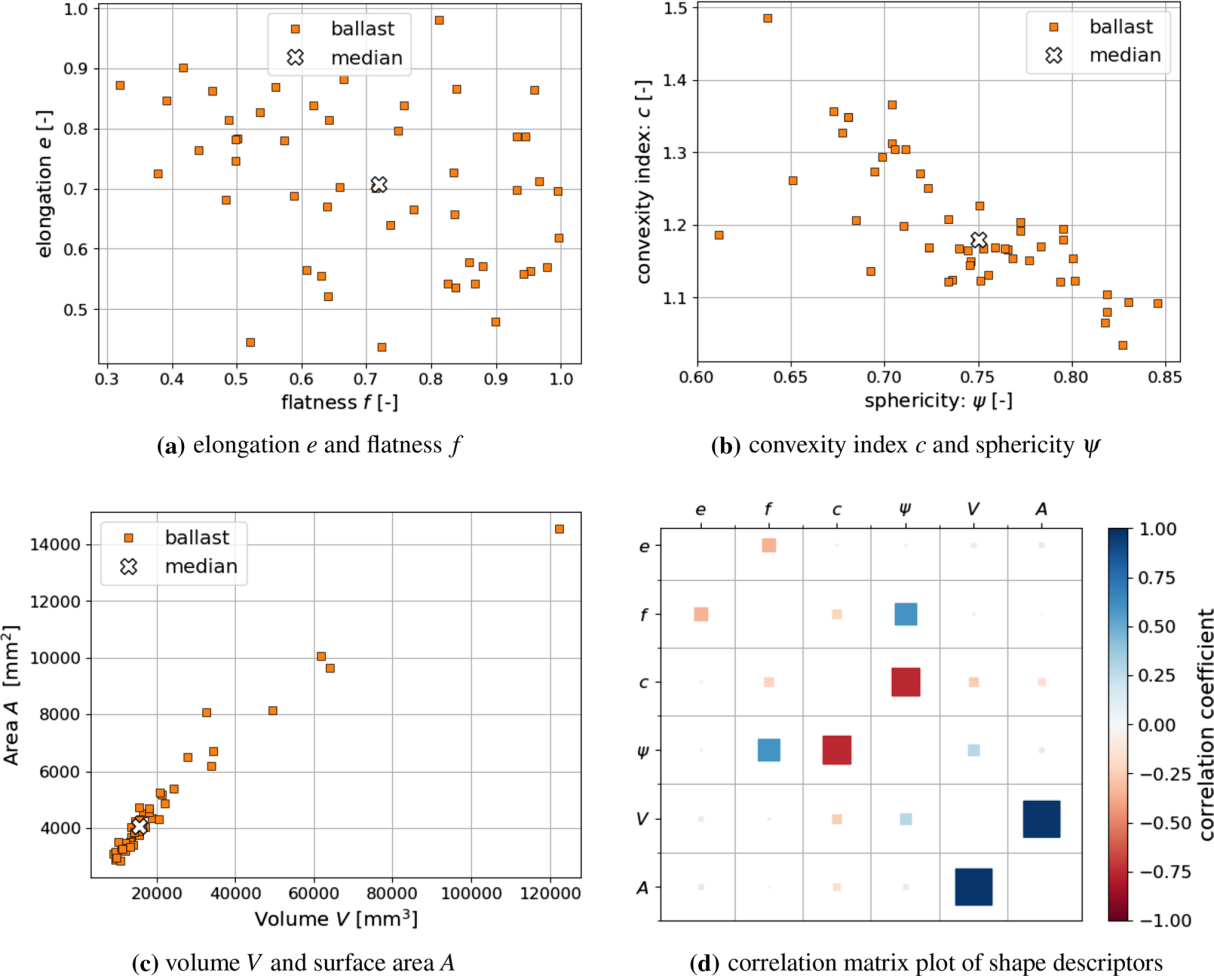
Shape descriptors of analysed ballast stones, compare [51]. Results for both types of ballast, Calcite and Kieselkalk, are presented together, as no clear distinction between both materials could be found

In [51], a shape analysis of the same two types of ballast, Calcite and Kieselkalk, was conducted using 25 stones each for 3D scanning (data openly available, [52]). Well-established shape descriptors, such as elongation, *e*, flatness, *f*, sphericity, $\psi$, convexity index, *c*, were evaluated. These shape descriptors are defined below in Eq. (1), denoting by *L*, *I*, *S* the longest, intermediate and shortest axes of the particle’s minimum bounding box, *V* the particle’s volume and *A* the particle’s surface.

1a $$\begin{aligned} e&=I/L \end{aligned}$$

1b $$\begin{aligned} f&=S/I\end{aligned}$$

1c $$\begin{aligned} \psi&= \root 3 \of {36 \pi V^2} / A\end{aligned}$$

1d $$\begin{aligned} c&= V(\text{ convex } \text{ hull }) / V(\text{ particle }) \end{aligned}$$

Regarding these shape descriptors no difference could be seen between both types of railway ballast: Calcite and Kieselkalk.

Moreover, three different angularity indices were compared in analytic tests, application to scanned data of sharp stones as well as artificially smoothed versions of the scanned stones. Only a newly introduced angularity index gave reasonable results in all considered cases, the scaled Willmore energy. The analysis of angularity always needs to clean scanned meshes from roughness information, which was done via mesh simplification. The calculated angularity values depend strongly on the level of mesh simplification. Thus, a comparison of scans of both types of ballast is possible, but no absolute value for the angularity of the ballast can be obtained. Moreover, clumps of spheres will be used as DEM shapes and here the calculation of angularity makes no sense. For these two reasons, angularity will not be considered in this work.

In Fig. 1 the shape descriptors of the scanned ballast stones are summarised. As no difference could be seen between Calcite and Kieselkalk, the corresponding values are merged. Shown are elongation *e* and flatness *f*: Fig. 1a, convexity index *c* and sphericity $\psi$: Fig. 1b, volume *V* and surface area *A*: Fig. 1c. Figure 1d shows a correlation matrix between the evaluated shape descriptors based on Pearson correlation coefficients. For improved visibility, the correlation of a quantity with itself is not plotted. Pearson correlation coefficients are sensitive to linear relations between two quantities. They range from $-1$ to $+1$, where $-1$ means total negative linear correlation, 0 means no linear correlation and $+1$ means total positive correlation. Non-linear correlations will be classified as low correlated by the Pearson coefficient. An additional visual inspection showed that this was not the case. The strongest correlation seen in the data is the one of *V* and *A*, which is also visible in Fig. 1c. Moreover, the correlation between *c* and $\psi$ is moderately established, as seen in Fig. 1b. All other quantities are low or not correlated at all. The shape descriptors and correlations of the ballast stones will be used for the shape modelling later on.

### Clump construction principals

**Fig. 2:**
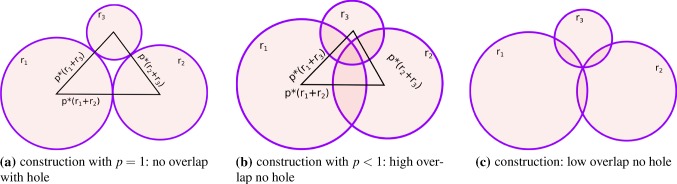
Clump construction principles

**Fig. 3:**
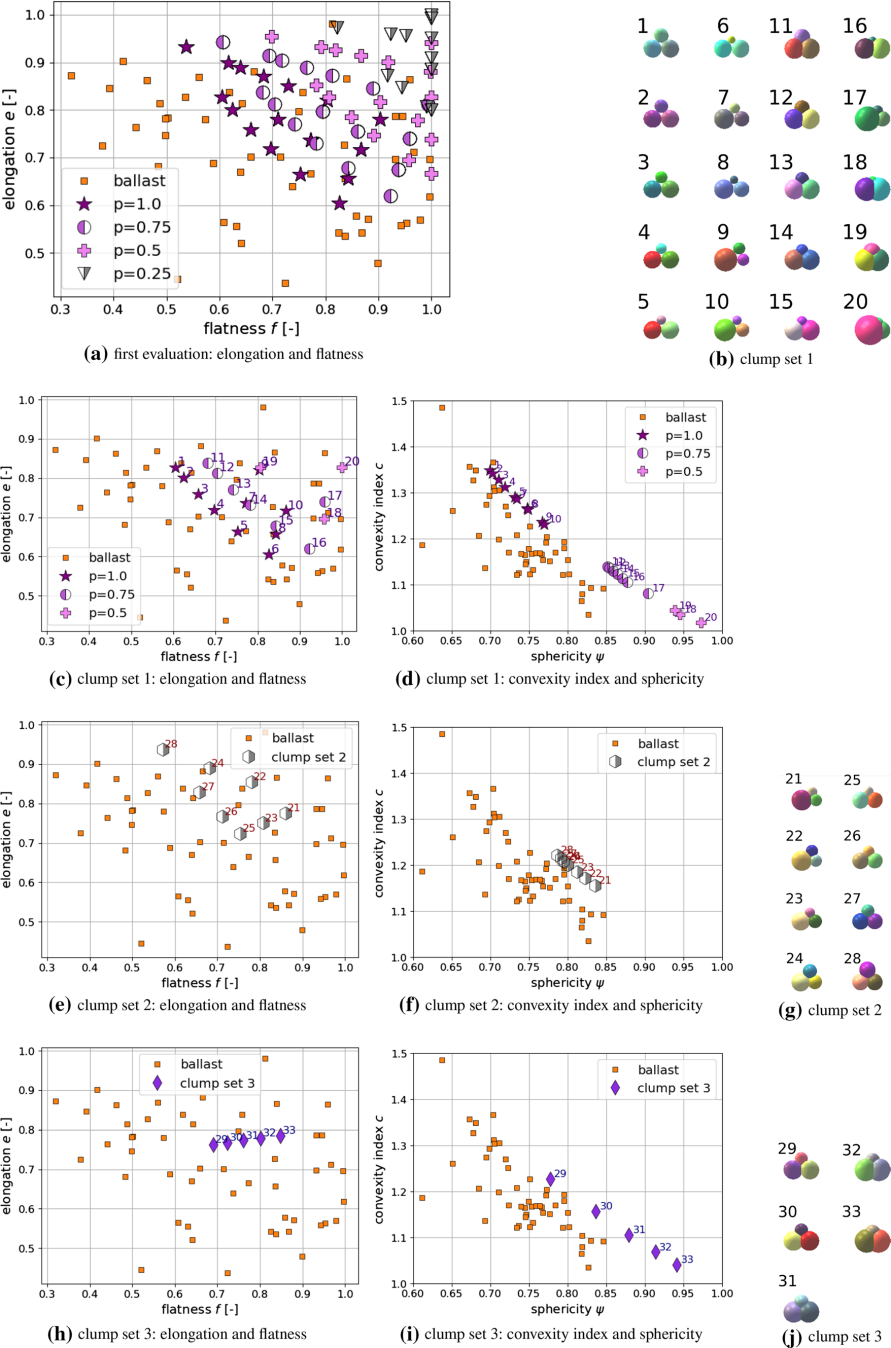
Construction of clumps

To construct a clump, at first one has to chose the number of spheres, which will build the clump. Clumps consisting of two spheres, are able to rotate freely along their longest axis. Moreover, these clumps have always an elongation of $S/I=1$. Therefore, in this work clumps of three spheres will be considered. They do not allow free rotations and it will be seen later in this section that they can approximate elongation, flatness, convexity and sphericity values of real ballast stones reasonably well. The clumps are described by the three different radii belonging to their sphere members and an overlap parameter *p*, see Fig. 2. In Fig. 2a, parameter $p=1$ and the resulting clump has no overlap but includes a hole. Contrasting, in Fig. 2b, *p* is much smaller than 1, closing the hole in the clump and resulting in high overlap. A compromise is shown in Fig. 2c, where clumps are constructed to intersect in exactly one point with small overlap. As discussed above, in the DEM simulations all particles will have the same size. The clumps are constructed to have all the same longest axis of $L=30~\hbox {mm}$.

These decisions made, the next step is to find clump shapes with elongation and flatness values similar to those of the measured ballast stones. Using the three sphere radii, $r_1, r_2, r_3$ and the overlap parameter *p*, the three axis *L*, *I*, *S* can be computed analytically. To figure out which elongation and flatness values are possible for this type of clump, a small computer script was written, where the sphere radii were varied for four different values of the overlap parameter *p*. In Fig. 3a the results of the computed elongation and flatness values are shown together with the results of the real ballast stones. The constructed clumps cover the upper right corner of the elongation and flatness plot, but cannot cover the less elongated (more columnar) ballast stones. From the constructed clumps shapes, 20 are chosen for further investigation, named clump set 1. The actual shape of these clumps can be seen in Fig. 3b and their corresponding elongation and flatness values in Fig. 3c. From these shapes also the convexity index *c* and the sphericity $\psi$ is evaluated and plotted in Fig. 3d. It is surprising that the ($\psi , c$) values of all clumps seem to lie on a straight line. Tests with clumps consisting of more spheres showed the same behaviour. However, this was not considered further in this work. The non-overlapping clump shapes with $p=1.0$, which include holes, show clearly higher convexity/lower sphericity values than those shapes which include overlapping i.e. $p=0.75$ or $p=0.5$. Between the non-overlapping and the overlapping clump shapes a gap can be seen. In this gap lie many of the values calculated from the ballast stones.

In a second step, clump shapes with low overlap and without hole will were constructed, as it is expected that they will fill the already mentioned gap in the ($\psi , c$) plot. The three spheres, which build these clumps, do intersect in exactly one point, see Fig. 2c, and aim at a low overlap volume. The resulting clumps are named clump set 2 and their elongation and flatness, convexity index and sphericity as well as their shape can be seen in Fig. 3e–g, respectively. These clumps show similar elongation and flatness values than the clumps chosen before, except that they do not reach flatness values below 0.7. In Fig. 3f they perfectly close the mentioned gap between the clumps with $p=1.0$ and $p=0.75$.

In a last step, one clump shape is chosen and overlap parameter *p* is varied in finer steps to investigate its influence in more detail. Clump number 3 is constructed without overlap ($p=1.0$). Keeping the ratio between the three radii $r_1, r_2, r_3$ fixed, *p* is varied in the steps $p=0.9, 0.8, 0.7, 0.6, 0.5$. The evaluated elongation and flatness values are shown in Fig. 3h, where it shows that all clump shapes are positioned along a straight line starting from clump shape 3. The ($\psi , c$) values of these clump span almost over the whole range of the clumps constructed before, as it can be seen in Fig. 3i. The shapes of the clump set 3 are shown in Fig. 3j.

### Further shape characterisation

A shape characteristic, which is not considered till now, is particle angularity. Curvature based angularity indices, as considered in [51], do not make sense for clumps of spheres. The spheres have constant curvature and only at the intersecting lines/points a different curvature exist.

**Fig. 4:**
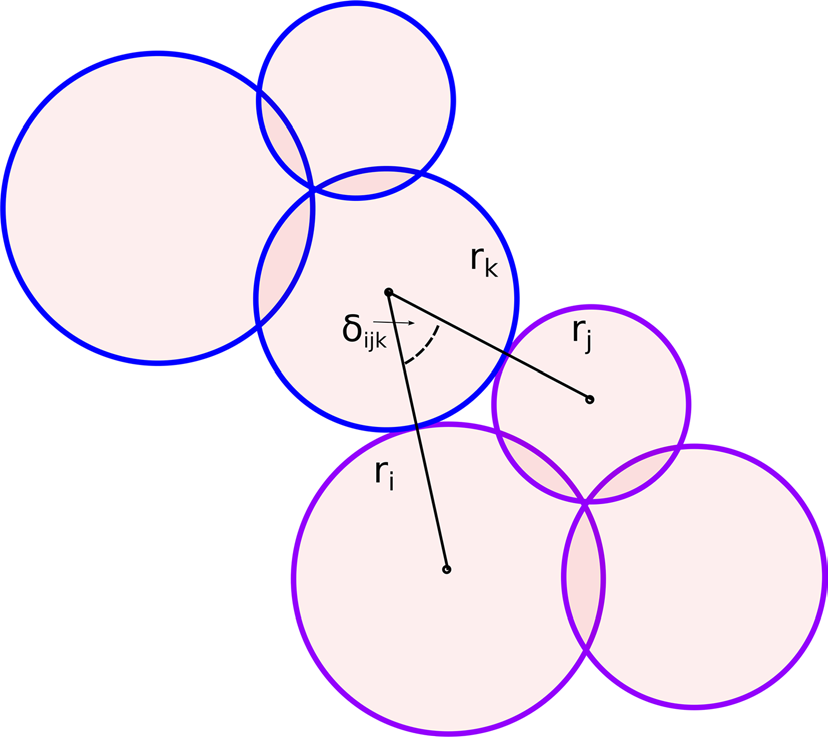
Draft of the concept of clump roughness angle $\delta _{ijk}$

To circumvent this problem, the concept of the “clump roughness angle” is introduced to quantify the before mentioned “geometric friction” of a clump. For its computation, two clumps are thought in contact, such that one sphere of the first clump contacts two spheres of the second clump, compare Fig. 4. The enclosed angle, $\delta _{ijk}$ can be calculated for all nine possible contacts. The clump roughness angle, $\delta$, is then defined to be the average of these nine values. This concept could be seen as an extension of the roughness angle introduced in [44], where it is used to characterise the wall (modelled by spheres) roughness in a shear flow.

**Fig. 5:**
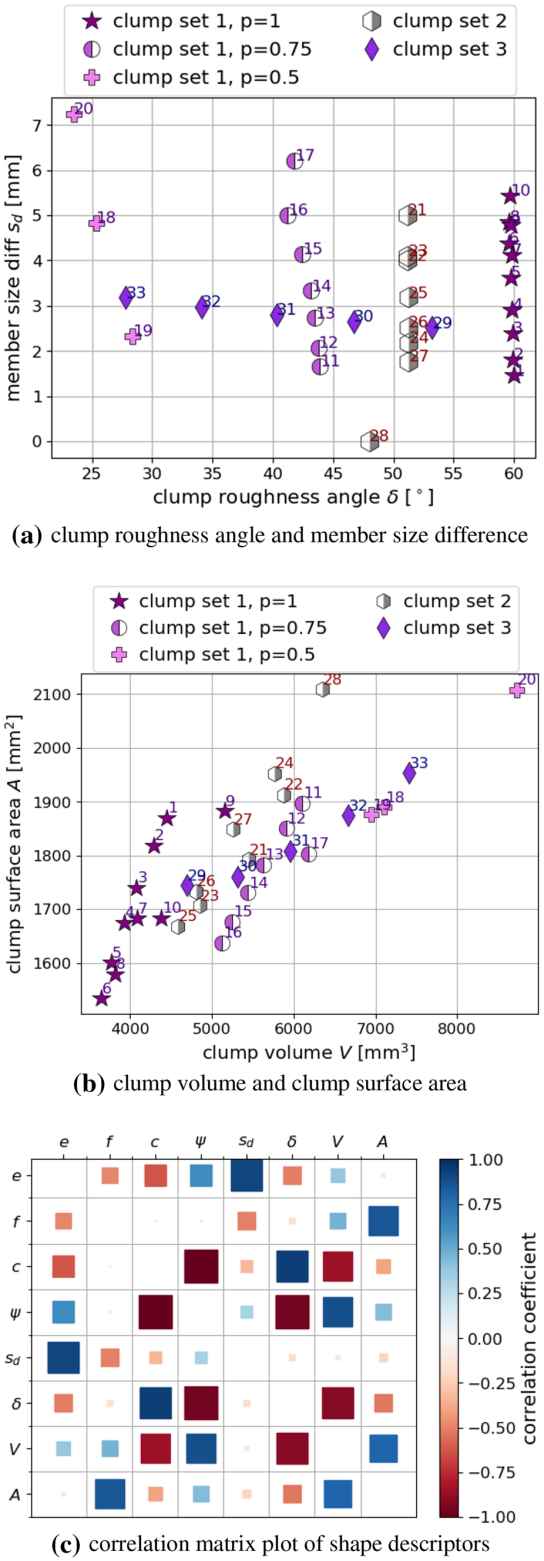
Further clump shape descriptors and correlation analysis

In addition to the clump roughness angle, the clumps are further characterised by the so called “member size difference”, $s_d$, which is the difference between the maximal and minimal radius of the spheres forming the clump. This quantity is expected to influence the packing behaviour. Fig. 5a shows the clump roughness angles, $\delta$, over the member size difference”, $s_d$, evaluated for all 33 constructed clump shapes. It can be seen that the overlap parameter *p* strongly influences the clump roughness angles $\delta$. The non-overlapping clumps have a clump roughness angle of 60$^\circ$ and with decreasing *p* also $\delta$ decays. Clumps, which are constructed with the same *p* have similar $\delta$ values. The member size difference, $s_d$, scatters in these groups. It’s extreme values are 0 for clump 28, composed of equi-sized spheres, and 7 mm for clump 20.

For completeness, also the clump volume *V* and the clump surface area is plotted in Fig. 5b. The clumps are constructed considerably smaller than the ballast stones, investigated in [51]. In the experiments conducted in [48], bigger stones were excluded due to size restrictions of the used test rig. Therefore, the clumps are also constructed smaller and a direct comparison to volume and surface area of ballast stones is not shown.

Figure 5c shows a correlation matrix plot using Pearson correlation coefficients. Again, additional visual inspection ensured that no non-linear correlations were misclassified by the Pearson coefficient. The correlations of the constructed clumps can be compared to the ones of the ballast stones, shown in Fig. 1d. The correlations between *c* and $\psi$ as well as between *V* and *A* are present for both ballast stones and the constructed clump shapes. The constructed clumps show further strong correlations between *V* and *c*, *V* and $\psi$, *A* and *f*. These additional correlations can be attributed to the clump construction method and the chosen size of *L*. The quantities $\delta$ and $s_d$ are specific shape descriptors for clumps of spheres and thus cannot be compared to the real ballast stones. For these quantities, strong correlations can be seen between $s_d$ and *e*, $\delta$ and *c*, $\delta$ and $\psi$, $\delta$ and *V*. This correlation analysis will be useful, when the simulation results discussed later will be linked to the clump’s shape descriptors.

## DEM simulation details

For all DEM simulations in this work the software YADE [56] will be used. It is Open-Source and utilises the soft contact approach together with explicit integration in time. Within this work the Hertz-Mindlin contact law will be used, which contains three material parameters: Young’s modulus, *E*, Poisson ratio, $\nu$, and friction coefficient, $\mu$. In previous works of the authors, [48, 49], it was pointed out that the Hertz-Mindlin contact law together with simple clump shapes is not sufficient to simulate an uniaxial compression and a direct shear test with only one set of material parameters. This problem was solved by using a different contact law: the conical damage model first introduced in [15]. The conical damage model takes into account additional physical effects such as edge breakage and thus introduces two additional material parameters. In the current work, focus is laid on the effect of particle shape on the packing behaviour. Therefore, the more simple Hertz-Mindlin contact law is used and the material parameters will be the same for all clump shapes, compare Table 1.

As already mentioned in the introduction of this work, it is important to address the calculation of mass and inertia tensor of clumps consisting of overlapping spheres. The YADE software deals with this topic as follows, see [55]:

- For non-overlapping clump members the clump’s mass $m_c$ is summed over members, the inertia tensor $I_c$ is computed using the parallel axes theorem: $I_c=\sum _i (m_i d_i^2+I_i)$, where $m_i$ is the mass of clump member *i*, $d_i$ is the distance from centre of clump member *i* to clump’s centroid and $I_i$ is the inertia tensor of the clump member *i*.
- For overlapping clump members the clump’s mass $m_c$ is summed over cells using a regular grid spacing inside axis-aligned bounding box of the clump, the inertia tensor is computed using the parallel axes theorem: $I_c=\sum _j (m_j d_j^2+I_j)$ , where $m_j$ is the mass of cell *j*, $d_j$ is the distance from cell center to clump’s centroid and $I_j$ is the inertia tensor of the cell *j*.

Further details on integration of force and motion for clumps of spheres are also given in [55].

**Table 1:** Material parameters used in DEM simulations

	*E* (GPa)	$\nu (-)$	$\mu (-)$	$\rho$ ($\hbox {kg/m}^3$)
Ballast	30	0.2	$0.01\le \mu \le 0.8$	2660.00
Steel box	200	0.28	0.5	7833.34

### Specimen generation and pre-compaction method

**Fig. 6:**
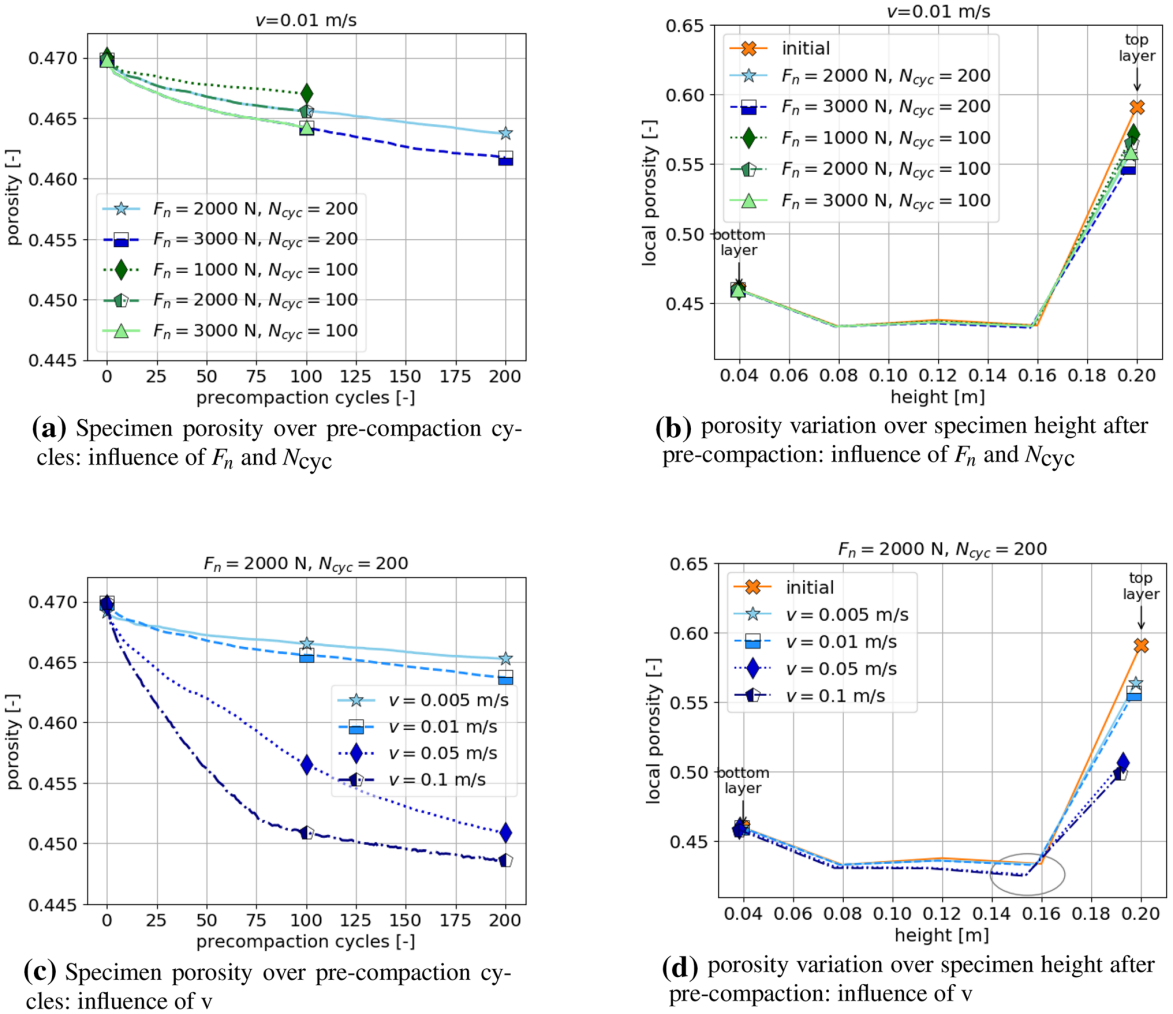
Influence of pre-compaction parameters on specimen porosity. Shape 7, $\mu =0.8$

All packings will be generated to fit in a steel box of the size $300 \text{ mm } \times 300 \text{ mm } \times 200 \text{ mm }$. A loose cloud of clumps is generated over the steel box and is allowed to settle under gravity. The interparticle friction coefficient, $\mu$, is varied in this initial phase of the calculation to generate packings of different porosities. When the packing has settled, all clumps are removed, which are not fully inside the box. As already said, the generated packings should be used in future work as a starting point for uniaxial compression and direct shear tests. Therefore, it is not enough to fill the box, but a pre-compaction is needed to avoid unrealistic settlement results in the uniaxial compression test. In the experiments, reported in [48], a vibrator compactor was used to compact the ballast specimen. In the simulations, a similar but less computationally expensive approach is chosen.

It is important to keep in mind the influence of the specimen generation and pre-compaction method, as they will affect the simulation results obtained with these samples. In [20] different shapes of clumps of spheres were used and two ways of specimen generation were compared. In one approach only the friction coefficient during the filling was modified. In a second approach cyclic simple shear was applied. Specimen of comparable porosities showed different coordination numbers. In a simulated direct shear test, the resulting initial slope differed, while after a small shear path the results were similar.

For the pre-compaction in this work, a plate is inserted above the specimen. The plate moves downwards with a constant velocity, $\hbox {v}$. When a given normal force, $F_{\mathrm{n}}$, is reached, the velocity is reversed until the plate is fully unloaded. This process is repeated for a given number of pre-compaction cycles, $N_{\mathrm{cyc}}$. The described process includes three parameters, $\hbox {v}, F_{\mathrm{n}}, N_{\mathrm{cyc}}$, whose influence on the packing will be investigated next.

In Fig. 6a the influence of $F_{\mathrm{n}}$ and $N_{\mathrm{cyc}}$ on the specimen porosity is shown, while the velocity $\hbox {v}=0.01~\hbox {m/s}$ is kept constant and $\mu =0.8$. Obviously, higher forces $F_{\mathrm{n}}$ and a higher number of pre-compaction cycles lead to denser samples, with the loosest sample with $\phi =0.467$ and the densest sample with $\phi =0.462$. More insight of the process is gained in Fig. 6b, where the porosity of the sample is calculated in five horizontal layers and plotted over sample height. Before pre-compaction, the initial porosity of the sample is quite constant in the middle of the sample, slightly higher at the bottom and clearly increased at the top layer. After the pre-compaction, the porosity is changed only in this top layer, which is seen as a step towards a more uniform porosity.

In a second step, the velocity $\hbox {v}$ is varied while the force $F_{\mathrm{n}}$ and number of pre-compaction cycles $N_{\mathrm{cyc}}$ are fixed. Within the chosen range, the influence of $\hbox {v}$ on the porosity is much greater then the one of $F_{\mathrm{n}}$ and $N_{\mathrm{cyc}}$, compare Fig. 6c. While $\hbox {v}=0.01$ m/s results in a sample with $\phi =0.464$, with $\hbox {v}=0.05$ m/s reduces to $\phi =0.451$. This strong influence can also be seen in Fig. 6d. It shows, that the higher velocities $\hbox {v}=0.05$ m/s and $\hbox {v}=0.1$ m/s decrease the porosity in the three upper layers of the sample. This is considered unrequested in this work, but different choices are possible of course. For all considered variants the coordination number of the resulting packings is very similar (between 5.5 and 5.75).

For the rest of this work, the following values will be used for pre-compaction, $F_{\mathrm{n}}=2000~\hbox {N}$, $N_{\mathrm{cyc}}=200$ and $\hbox {v}=0.01~\hbox {m/s}$. In checks not shown here, it was ensured that the obtained packings give realistic settlements in uniaxial compression tests.

## Analysis of packing behaviour

With the previously defined clump shapes and the pre-compaction method at hand, the generation of packings can be simulated. For each of the chosen 33 clump shapes, simulations are run using three different values for the friction coefficient: $\mu =0.01, 0.45, 0.8$. With $\mu =0.01$ (nearly) the densest possible packing is obtained, while $\mu =0.8$ is a rather high value for railway ballast friction coefficients, compare [23] or [42], giving loose packings. Thus, for each considered clump shape dense, intermediate and loose packings will be studied to be able to separate the effects of clumps shape and material parameters, i.e. friction coefficient. The influence of the shape descriptors on the obtained packing properties will be discussed in the first subsection. In the second subsection, the choice of clumps for modelling the given type of railway ballast will be addressed.

### Shape descriptors and packing characteristics

**Fig. 7:**
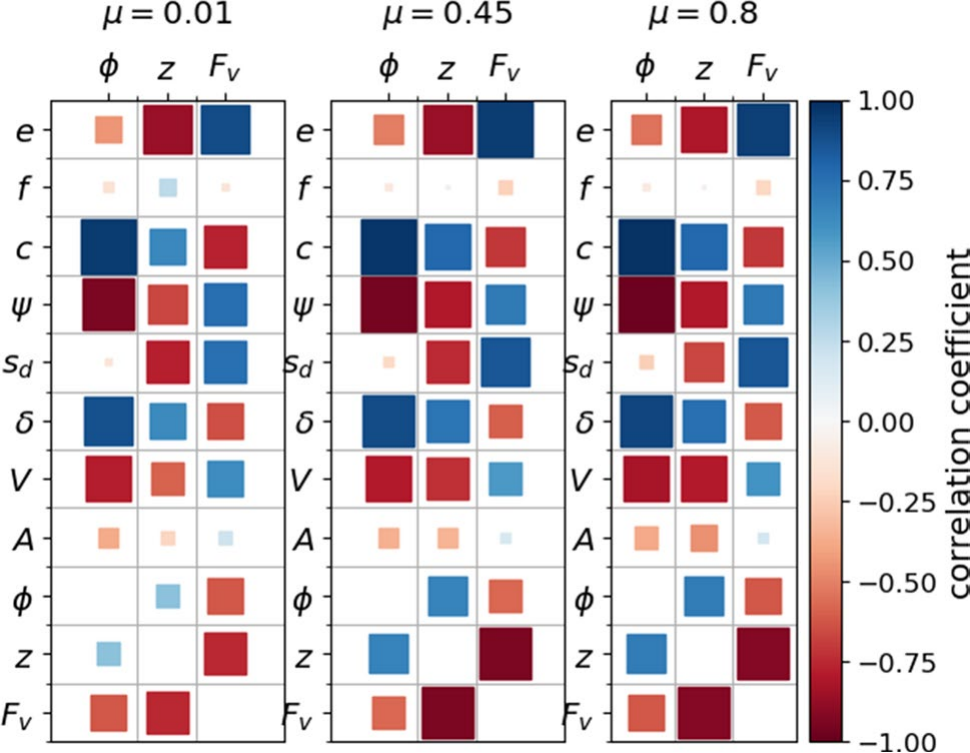
Correlation analysis for porosity $\phi$, coordination number *z* and isotropic fabric $F_v$

The simulated packings will be characterized by porosity, coordination number and fabric. The effect of the clump’s shape (descriptors) on these quantities will be discussed. Correlations are sought in simulations results using three different values of interparticle friction coefficients $\mu =$ 0.01, 0.45, 0.8. In this way, the influence of particle shape and used material parameters are separated. As a first step, Fig. 7 shows the correlation matrix plot between the shape descriptors on one axis and porosity $\phi$, coordination number *z* and fabric $F_v$ on the other axis. In the lower part of the plot, also the correlations of $\phi$, *z* and $F_v$ with each other is shown. Analogously as in Fig. 1d, Pearson correlation coefficients are calculated, which are sensitive only for a linear relationship between two quantities. Non-linear relations will be classified as low correlated by the Pearson index. An additional visual inspection showed that this was not the case for the given data.

In Fig. 7, it can be seen that the porosity $\phi$ correlates strongly with *c*, $\psi$, $\delta$ and *V*. The correlations are nearly constant for all three different values interparticle friction coefficients $\mu =0.01, 0.45, 0.8$. The correlation between *c* and $\phi$ is in accordance with the intuitive understanding that nearly convex clumps (e.g. shape 20) will pack more densely than clumps with high convexity index (e.g. shape 1). From the analysis in Sect. 2.3, it is known that *c* correlates strongly with $\psi$, $\delta$ and *V*. Summarising the found correlations, loose packings are formed by clumps with high convexity index, low sphericity, high clump roughness angle and low clump volume. Dense packings are formed by clumps with opposite characteristics.

Examining again Fig. 7, the correlations found for the coordination number *z* are higher in number but less pronounced than the ones found for the porosity. While the correlations between *z* and $c, \psi , \delta , V$ increase with increasing $\mu$, the correlations to *e* and $s_d$ slightly weaken with increasing $\mu$. The correlation between the porosity $\phi$ and coordination number *z* is weak for $\mu =0.01$ but is quite pronounced for the two higher $\mu$ values.

As third packing characterisation, the isotropic fabric $F_v$ is considered. For the calculation of the fabric tensor *F* the definition in [22] is used

2 $$\begin{aligned} F=\frac{1}{V_A} \sum _{p \in V_s} V_p \sum _{c \in p} n_c \otimes n_c\; , \end{aligned}$$

where $V_A$ is the averaging volume, $V_p$ is the volume of particle *p* in $V_A$, and $n_c$ is the normal unit branch vector from the centre of particle *p* to contact *c*. This definition of the fabric tensor takes into account different particle sizes, which is in contrast to the frequently used fabric tensor definition

3 $$\begin{aligned} F_0=\frac{1}{N_c} \sum _{c \in N_c} n_c \otimes n_c\; , \end{aligned}$$

where $N_c$ is the total number of contacts in the packing. From the fabric tensor as defined in Eq. 2 the isotropic fabric $F_v$ is defined, which can be considered as a measure of the strength of the contact network:

4 $$\begin{aligned} F_v={{\,\mathrm{tr}\,}}(F) \; . \end{aligned}$$

Going back to Fig. 7, it can be seen that the isotropic fabric $F_v$ is correlated to all shape descriptors, which show correlations to *z*. While the correlations of $F_v$ to $c, \psi , \delta , V$ slightly decrease with increasing $\mu$, the correlations to $e, s_d$ slightly increase with increasing $\mu$.

Fig. 7, shows a strong correlation between the isotropic fabric $F_v$ and *z* and weak correlation to $\phi$. This finding is not surprising, as for packings composed of spheres a linear relation can be stated, see [22].

### Choice of clumps shapes for the given material

From the investigated clump shapes, those have to be chosen which can be used to model the two types of ballast considered, Calcite and Kieselkalk. In [48] these ballast types showed porosities between $0.42\le \phi \le 0.46$ in the experiments. Thus, all clump shapes, whose simulated porosity lies within the specified range (for any of the used $\mu$ values), will be considered a possible candidate for modelling this ballast. Further steps for the reduction of possible clumps shapes will be discussed later.

**Fig. 8:**
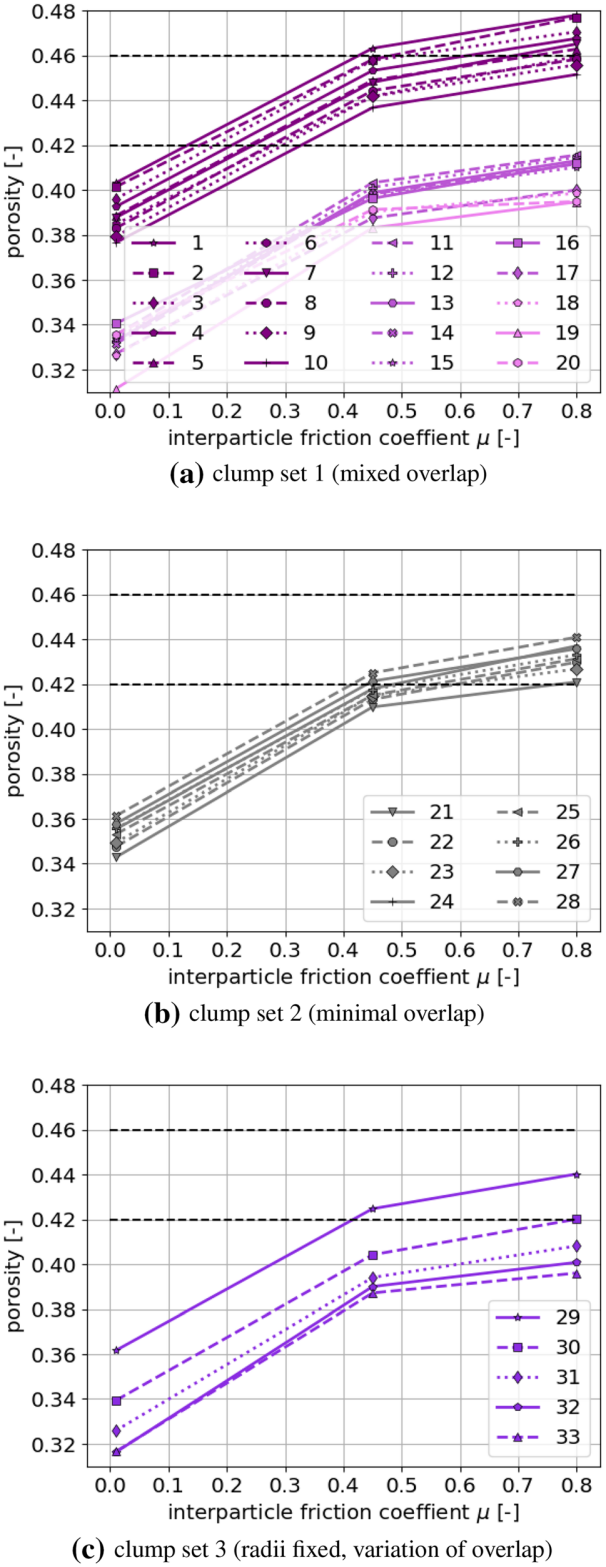
Porosity of samples generated with the different clumps, dependency of interparticle friction coefficient $\mu$. The horizontal dashed lines show the range of experimental porosities measured in [48]

The obtained porosities are plotted over the friction coefficient $\mu$ in Fig. 8 for the three different clump sets. Each simulation was repeated two times to check how much the results scatter with the randomly chosen initial position of the particles. In these repetition simulations, the obtained porosities show surprisingly low scattering with maximal differences of porosities 0.005. The scatterings is similar for all 33 clump shapes considered. The dashed lines in Fig. 8 show the range of experimental porosities measured in [48]. From the results, big differences in the packings’ porosity can be seen. In Fig. 8a, the highest porosities, ranging from 0.38 till 0.48, are shown from shapes 1 till 10, which are constructed with $p=1$, i.e. non-overlapping including a hole in the clump. Clumps shapes 11 till 17 belong to $p=0.75$ and a gap in the porosity values can be seen, as the obtained values lie between 0.33 and 0.42. Shapes 18 till 20 have with $p=0.5$ the lowest porosities. The loosest packing of these shapes is only little looser than the densest packing of the non-overlapping shapes. In Fig. 8b, clump set 2 is constructed to have small overlapping volume and no hole. The obtained porosities lie quite exactly between those of clumps 1 till 10 with $p=1$ and the clumps 11 till 20 with $p\le 0.75$. The influence of parameter *p* on the porosities can be seen best in Fig. 8c, where $0.5\le p \le 0.9$. Decreasing parameter *p* means increasing overlap and this results in denser packings. For shape 32 with $p=0.6$ and shape 33 with $p=0.5$ almost the same porosities are obtained.

**Fig. 9:**
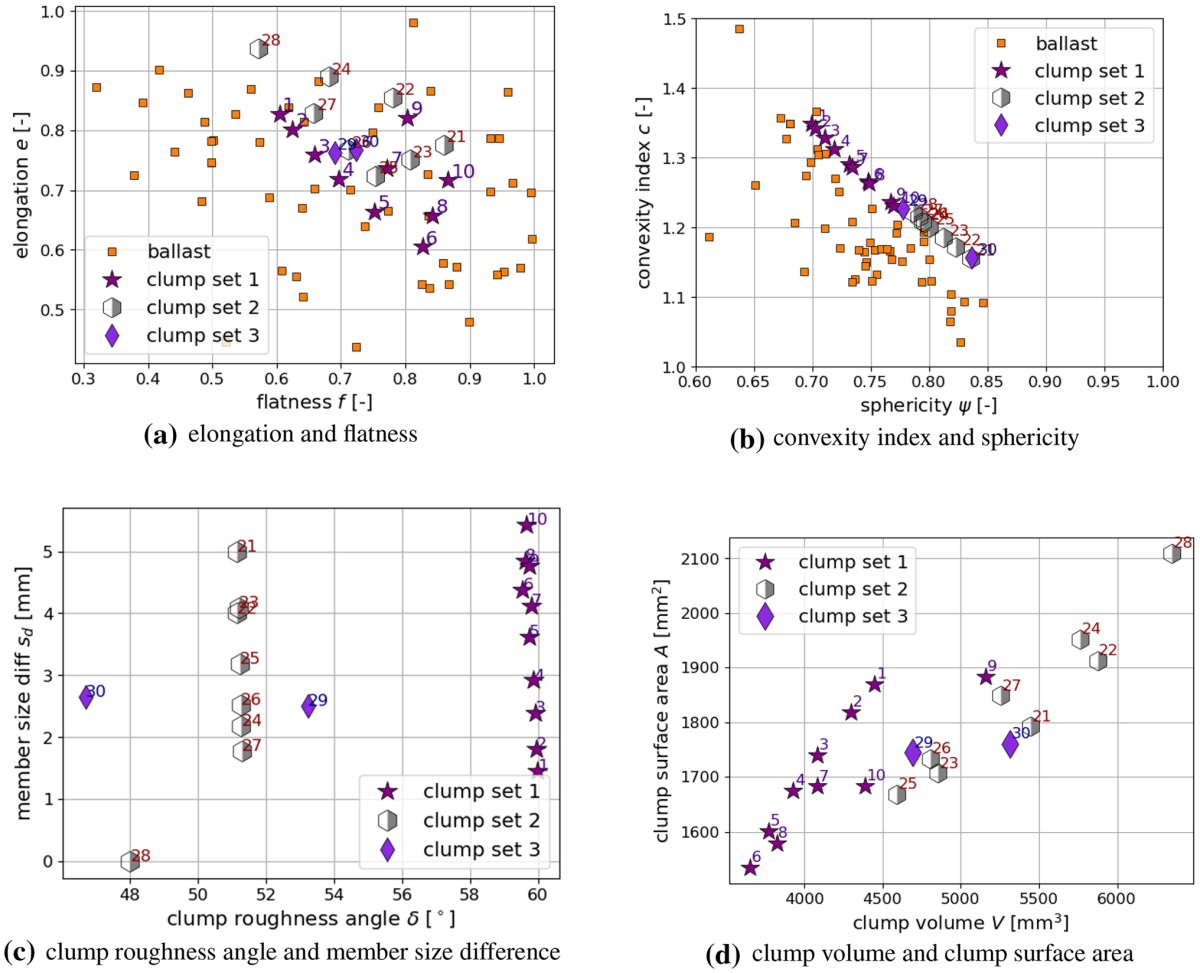
Shape descriptors of the chosen clumps, compared to those real ballast stones when applicable

**Fig. 10:**
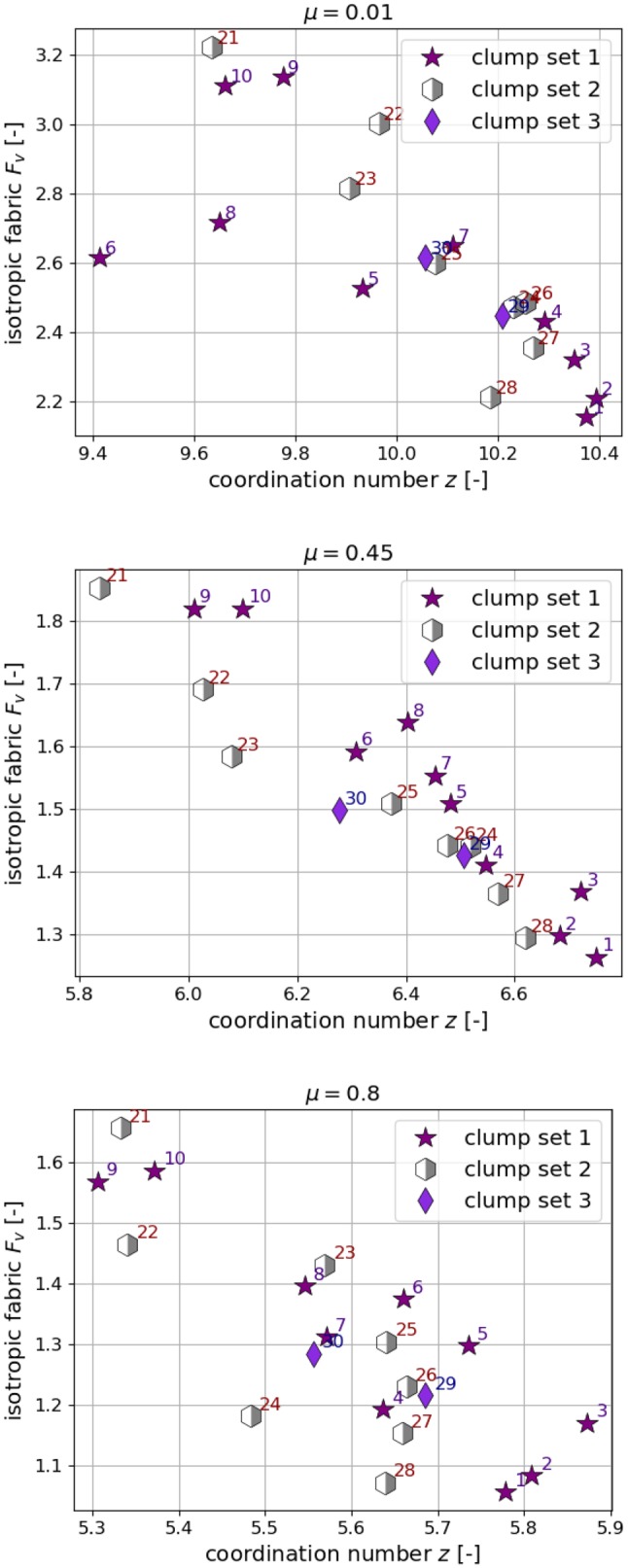
Coordination number and isotropic fabric for all three values of $\mu$

Possible clump shapes for the simulation of the two types of ballast used in [48], Calcite and Kieselkalk, are those with porosities in the searched experimental range:

- from clump set 1: shapes **1 to 10**
- from clump set 2: shapes **21 to 28**
- from clump set 3: shapes **29 and 30**

Further insight in shape modelling can be gained, from the shape descriptors of the chosen clumps plotted in Fig. 9, when possible together with those of the real ballast stones. Elongation and flatness values of the real ballast stones cover a wide range of values in Fig. 9a. The chosen clumps also cover quite a big part of their possible range due to clump construction, compare Fig. 3a. This is not surprising, as neither elongation nor flatness show a strong correlation with $\phi$, which was used to choose the clump shapes. In Fig. 9b, *c* and $\psi$ of the chosen clumps are plotted together with the values of the real ballast stones. Due to the strong correlation of the porosity $\phi$ with *c* and $\psi$, all chosen clumps have $c\ge 1.15$ and $\psi \le 0.84$, while all rejected clumps have smaller *c* and larger $\psi$ values. The range of $(c, \psi )$ values of the clump shapes matches well with those of the real ballast stones. Thus, when a different material is to be modelled via simple particle shapes, matching the convexity and sphericity might be a good first step. In Fig. 9c, clump roughness angle $\delta$ and member size difference $s_d$ are plotted. Both quantities are specific to the clump modelling and therefore no comparison to the ballast stones is possible. While the $s_d$ values of the chosen clumps cover nearly the complete range, it is apparent that the chosen clumps have all $\delta \ge 45^\circ$ and all rejected clumps have smaller $\delta$ values. This result is expected, as $\delta$ correlates strongly with the porosity $\phi$ (which was used to choose the clumps) but $s_d$ does not show this correlation with $\phi$. Finally, Fig. 9d shows the volume and surface area of the chosen clumps. Although *V* shows a strong correlation with the porosity, chosen clump shapes and rejected ones cannot be separated by volume. This is in contrast to the other shape descriptors, which do correlate strongly with the porosity, i.e. $c, \psi , \delta$. The volume and surface area of the clumps is smaller than the ones of the real stones, due to DEM modelling conventions, i.e. longest size of the particle and sample size have a ratio of 10. Therefore no comparison is shown.

For a further analysis of similarities and differences between the chosen clump shapes, Fig. 10 shows coordination number *z* and isotropic fabric $F_v$ for three values of $\mu$. In the plots, the chosen clump shapes vary in coordination number by approximately 1 and in isotropic fabric by approximately 0.7 (depending an the $\mu$ value used). From the conducted repetition simulations, the maximal difference in the calculated coordination number is 0.23 and in isotropic fabric is 0.07. Thus, the scattering caused by the particles’ initial position is with 10% for $F_v$ and $20\%$ for *z* much larger than the scattering observed in the porosities. Nevertheless, the correlation between *z* and $F_v$ is well established and most shapes keep their place on the “correlation line” for all three $\mu$ values, i.e. shapes 1, 2, 3 have high *z* values and low $F_v$ values while shapes 21, 9, 10 have low *z* values and high $F_v$ values.

More conclusions can be drawn from the information gathered so far. Clump shapes with similar $(c, \psi )$ values result in specimens with similar porosities, due to the strong correlation between $c,\psi ,\phi$. This is the case for shapes 21 and 30, who have similar $(c, \psi )$ but all other shape descriptors differ. The resulting packings show quite different results for *z* and $F_v$, compare Fig. 10, such that a different response under loading can be expected. This will be of importance in the future work of choosing suitable clump shapes and parametrising the contact model.

## Conclusions and outlook

In this work, simple and computationally efficient DEM shape models for two types of railway ballast are constructed. The novelty of the presented approach lies in the approximation of known shape descriptors of this ballast, [51], skipping the idea of actual visual similarity between real grains and DEM shapes. While the angularity of railway ballast is well addressed in the literature, the stones’ non-convexity is rarely mentioned or quantified. Both properties can be expected to influence the bulk material behaviour. In current DEM codes angular shapes are represented by polyhedra, which cannot deal (efficiently) with non-convexity. In contrast to this, clumps of spheres cannot model angularity, but easily build up non-convex shapes. When clumps are constructed of few spheres, they are highly computationally efficient and they are available in many DEM codes.

Clumps consisting of three spheres are constructed to approximate the railway ballast’s flatness, elongation, convexity index and sphericity. Clumps of spheres cannot be angular, but spheres can be arranged to provide high “surface roughness” [46] or “geometric friction” [43] and [19] to use nomenclature from the literature. To quantify this property the so called “clump roughness angle”, $\delta$, is introduced. Correlations between the clump’s shape descriptors are compared to those between the ballast stones shape descriptors. The ballast stones show only two strong correlations: between convexity index *c* and sphericity $\psi$ and between surface area *A* and volume *V*. The correlations are also present in the constructed clumps’ shape descriptors, but additional correlations exist as well due to the clump construction method.

DEM simulations of the packing behaviour are conducted for each clump shape. The usage of three different values of the interparticle friction coefficient $\mu$ results in loose, intermediate and dense packings. These simulated packings are characterised by their porosity $\phi$, coordination number *z* and isotropic fabric $F_v$. The relation between particle shape (descriptors) and packing (characteristic) is investigated in a correlation analysis. The porosity $\phi$ is strongly correlated with $c, \psi , \delta , V$, for all three values of $\mu$ used. These four shape descriptors are highly correlated among themselves for the constructed clumps. Thus, the control of one of them already gives a good correlation to the obtained porosities.

The packing’s coordination number and isotropic fabric correlate with the same shape descriptors. These correlations are weaker, but involve more shape descriptors. The found correlations are influenced by $\mu$ in opposing trends for *z* and $F_v$ . Also, *z* and $F_v$ correlate with each other. Thus, coordination number and isotropic fabric are related to particle shape in a much more complex way than the packing’s porosity.

With these relations clarified, the simulated packing porosities are compared to experimental results obtained in [48]. Clumps with porosity values in the experimental range, are considered possible candidates for the simulations of the two ballast types. Comparing the shape descriptors of these chosen clumps to the ones of the ballast stones, gives a good visualisation of the results found so far. With $\phi$ being the criterion of choice, elongation and flatness of the clumps are (mostly) unimportant: these quantities are uncorrelated to the porosity. In contrast, convexity index and sphericity of chosen clumps and ballast stones are in good agreement: these quantities show strong correlations to the porosity. Thus, to derive simple shape models of a given particle shape, matching one of these shape descriptors, might be good first step to bring simulated porosities closer to measured ones.

In future work, the generated packings will be used as a staring point for the simulation of uniaxial compression and direct shear tests. In comparison with available experimental data, the calibration of material parameters (Young’s modulus, friction coefficient) will be addressed (separately for each clump shape). Possible speed ups of this lengthy procedure, involving measured material parameters will be investigated. In this way, the number of clumps shapes possible for modelling the used ballast types will be further reduced. Using additional measurements for more complex loading cases will be used to further validate the developed DEM models.

This work deals with the DEM simulation of railway ballast, but the underlying idea is a general one. In the simulation of granular material consisting of complex shaped particles, focus is often laid on a precise shape modelling in DEM. These highly computational shape models are frequently combined with very simple models for the contact forces, e.g. linear spring models. The authors of this work propose a so-called balanced approach. Particle shape is modelled as simple as possible and combined with more advanced contact laws, if needed. In this work, clumps of three spheres are constructed to approximate known shape descriptors, skipping the idea of actual visual similarity between real grains and DEM shapes. This approach might also be suitable for the DEM simulation of other applications involving complex shaped particles, e.g. in pharmaceutical, geotechnical or additive manufacturing industry. For different simulated applications a careful analysis of the considered shape descriptors will be necessary – it can be expected that different particle shape descriptors are relevant for modelling a hopper discharge and the mechanical behaviour of densely compacted railway ballast. DEM models using simple clusters of few clumped spheres have the potential to reduce computational costs drastically. Dependent on the simulated application, it should be carefully checked, if the used contact law includes all relevant physical effects. As always, a thorough validation of such a DEM model is necessary.

## Electronic supplementary material

Below is the link to the electronic supplementary material.

Online Resource 1 (ESM_1.csv) Details on all clumps constructed in this paper: including size and positions of spheres forming the clump and values of all computed shape descriptors. (6 KB)

## Data Availability

All data generated during this study are included in this published article and its supplementary information file.

## References

[1] Ahmed S, Harkness J, Le Pen L, Powrie W, Zervos A (2016). Numerical modelling of railway ballast at the particle scale. Int. J. Numer. Anal. Methods Geomech..

[2] Al-Rousan T, Masad E, Tutumluer E, Pan T (2007). Evaluation of image analysis techniques for quantifying aggregate shape characteristics. Constr. Build. Mater..

[3] Bagheri G, Bonadonna C, Manzella I, Vonlanthen P (2015). On the characterization of size and shape of irregular particles. Powder Technol..

[4] Blott SJ, Pye K (2008). Particle shape: a review and new methods of characterization and classification. Sedimentology.

[5] Bullard JW, Garboczi EJ (2013). Defining shape measures for 3d star-shaped particles: sphericity, roundness, and dimensions. Powder Technol..

[6] Chen C, Indraratna B, McDowell G, Rujikiatkamjorn C (2015). Discrete element modelling of lateral displacement of a granular assembly under cyclic loading. Comput. Geotech..

[7] Coetzee C (2016). Calibration of the discrete element method and the effect of particle shape. Powder Technol..

[8] Cundall PA, Strack ODL (1979). A discrete numerical model for granular assemblies. Geotechnique.

[9] Desu RK, Annabattula RK (2019). Particle size effects on the contact force distribution in compacted polydisperse granular assemblies. Granul. Matter.

[10] Ferellec JF, McDowell G (2010). Modelling realistic shape and particle inertia in dem. Geotechnique.

[11] Ferellec JF, McDowell GR (2010). A method to model realistic particle shape and inertia in dem. Granul. Matter.

[12] Gao R, Du X, Zeng Y, Li Y, Yan J (2012). A new method to simulate irregular particles by discrete element method. J. Rock Mech. Geotech. Eng..

[13] Garboczi E, Liu X, Taylor M (2012). The 3-d shape of blasted and crushed rocks: from 20 $$\mu $$m to 38 mm. Powder Technol..

[14] Garcia X, Latham JP, Xiang J, Harrison J (2009). A clustered overlapping sphere algorithm to represent real particles in discrete element modelling. Geotechnique.

[15] Harkness J, Zervos A, Le Pen L, Aingaran S, Powrie W (2016). Discrete element simulation of railway ballast: modelling cell pressure effects in triaxial tests. Granul. Matter.

[16] Huang H, Tutumluer E (2014). Image-aided element shape generation method in discrete-element modeling for railroad ballast. J. Mater. Civ. Eng..

[17] Indraratna B, Ngo N, Rujikiatkamjorn C, Vinod J (2014). Behavior of fresh and fouled railway ballast subjected to direct shear testing: discrete element simulation. Int. J. Geomech..

[18] Indraratna B, Thakur P, Vinod J (2010). Experimental and numerical study of railway ballast behavior under cyclic loading. Int. J. Geomech..

[19] Irazabal J, Salazar F, Onate E (2017). Numerical modelling of granular materials with spherical discrete particles and the bounded rolling friction model. Application to railway ballast. Comput Geotech.

[20] Katagiri J, Matsushima T, Yamada Y (2014). Variations in shear behavior among specimens with different packing patterns. Granul. Matter.

[21] Kozicki J, Tejchman J, Mróz Z (2012). Effect of grain roughness on strength, volume changes, elastic and dissipated energies during quasi-static homogeneous triaxial compression using DEM. Granul. Matter.

[22] Kumar N, Luding S, Magnanimo V (2014). Macroscopic model with anisotropy based on micro-macro information. Acta Mech..

[23] Kwan, C.C.J.: Geogrid reinforcement of railway ballast. Ph.D. thesis, University of Nottingham (2006)

[24] Laryea S, Baghsorkhi MS, Ferellec JF, McDowell G, Chen C (2014). Comparison of performance of concrete and steel sleepers using experimental and discrete element methods. Transp. Geotech..

[25] Le Pen M, Powrie W, Zervos A, Ahmed S, Aingaran S (2013). Dependence of shape on particle size for a crushed rock railway ballast. Granul. Matter.

[26] Lee JRJ, Smith ML, Smith LN, Midha PS (2005). A mathematical morphology approach to image based 3d particle shape analysis. Mach. Vis. Appl..

[27] Lu G, Third J, Müller C (2015). Discrete element models for non-spherical particle systems: from theoretical developments to applications. Chem. Eng. Sci..

[28] Lu M, McDowell G (2007). The importance of modelling ballast particle shape in the discrete element method. Granul. Matter.

[29] Lu M, McDowell G (2010). Discrete element modelling of railway ballast under monotonic and cyclic triaxial loading. Geotechnique.

[30] Masad E, Saadeh S, Al-Rousan T, Garboczi E, Little D (2005). Computations of particle surface characteristics using optical and x-ray ct images. Comput. Mater. Sci..

[31] McDowell GR, Li H (2016). Discrete element modelling of scaled railway ballast under triaxial conditions. Granul. Matter.

[32] Nie Z, Liang Z, Wang X (2018). A three-dimensional particle roundness evaluation method. Granul. Matter.

[33] Nie Z, Wang X, Liang Z, Gong J (2018). Quantitative analysis of the three-dimensional roundness of granular particles. Powder Technol..

[34] O’Sullivan C (2011). Particulate Discrete Element Modelling—A Geomechanics Perspective.

[35] Ouhbi N, Voivret C, Perrin G, Roux JN (2017). 3d particle shape modelling and optimization through proper orthogonal decomposition. Granul. Matter.

[36] Pan Tongyan, Tutumluer Erol, Anochie-Boateng Joseph (2006). Aggregate Morphology Affecting Resilient Behavior of Unbound Granular Materials. Transportation Research Record: Journal of the Transportation Research Board.

[37] Parteli EJR (2013). Dem simulation of particles of complex shapes using the multisphere method: application for additive manufacturing. AIP Conf. Proc..

[38] Pöschel T, Buchholtz V (1993). Static friction phenomena in granular materials: Coulomb law versus particle geometry. Phys. Rev. Lett..

[39] Pourtavakoli H, Parteli EJR, Pöschel T (2016). Granular dampers: Does particle shape matter?. New J. Phys..

[40] Qian Y, Lee S, Tutumluer E, Hashash Y, Mishra D, Ghaboussi J (2013). Simulating ballast shear strength from large-scale triaxial tests. Transp. Res. Rec..

[41] Qian Y, Lee SJ, Tutumluer E, Hashash YMA, Ghaboussi J (2018). Role of initial particle arrangement in ballast mechanical behavior. Int. J. Geomech..

[42] Quintanilla, I.D.: Multi-scale study of the degradation of railway ballast. Ph.D. thesis, Universite Grenoble Alpes (2018)

[43] Roth LK, Jaeger HM (2016). Optimizing packing fraction in granular media composed of overlapping spheres. Soft Matter.

[44] Schuhmacher Paul, Radjai Farhang, Roux Stéphane (2017). Wall roughness and nonlinear velocity profiles in granular shear flows. EPJ Web Conf..

[45] Schwager T, Pöschel T, Popp K, Schiehlen W (2003). Rigid body dynamics of railway ballast. System Dynamics and Long-Term Behaviour of Railway Vehicles, Track and Subgrade.

[46] Soltanbeigi B, Podlozhnyuk A, Papanicolopulos SA, Kloss C, Pirker S, Ooi JY (2018). Dem study of mechanical characteristics of multi-spherical and superquadric particles at micro and macro scales. Powder Technol..

[47] Stahl M, Konietzky H (2011). Discrete element simulation of ballast and gravel under special consideration of grain-shape, grain-size and relative density. Granul. Matter.

[48] Suhr B, Marschnig S, Six K (2018). Comparison of two different types of railway ballast in compression and direct shear tests: experimental results and dem model validation. Granul. Matter.

[49] Suhr B, Six K (2017). Parametrisation of a dem model for railway ballast under different load cases. Granul. Matter.

[50] Suhr B, Six K (2018). Compression tests and direct shear test of two types of railway ballast [data set]. Zenodo.

[51] Suhr, B., Skipper, W.A., Lewis, R., Six, K.: Shape analysis of railway ballast stones: curvature-based calculation of particle angularity. Submitted for publication 2019 10.1038/s41598-020-62827-w PMC7142084 32269233

[52] Suhr B, Six K, Skipper WA, Lewis R (2020). 3D scans of two types of railway ballast including shape analysis information [data set]. Zenodo.

[53] Sun Y, Indraratna B, Nimbalkar S (2014). Three-dimensional characterisation of particle size and shape for ballast. Geotech. Lett..

[54] Tutumluer E, Qian Y, Hashash YM, Ghaboussi J, Davis DD (2013). Discrete element modelling of ballasted track deformation behaviour. Int. J. Rail Transp..

[55] Šmilauer, V., Chareyre, B.: DEM formulation. In: Yade Documentation 2nd ed. The Yade Project (2015). 10.5281/zenodo.34044. http://yade-dem.org/doc/

[56] Šmilauer, V., et al.: Yade Documentation 2nd ed. The Yade Project (2015). 10.5281/zenodo.34073. http://yade-dem.org/doc/

[57] Wadell H (1932). Volume, shape, and roundness of rock particles. J. Geol..

[58] Wadell H (1933). Sphericity and roundness of rock particles. J. Geol..

[59] Wadell H (1935). Volume, shape, and roundness of quartz particles. J. Geol..

[60] Xiao J, Zhang D, Wei K, Luo Z (2017). Shakedown behaviors of railway ballast under cyclic loading. Constr. Build. Mater..

[61] Yang X, Chen S, You Z (2017). 3d voxel-based approach to quantify aggregate angularity and surface texture. J. Mater. Civ. Eng..

[62] Zhao B, Wang J (2016). 3d quantitative shape analysis on form, roundness, and compactness with $$\mu $$CT. Powder Technol..

[63] Zhao S, Zhou X, Liu W (2015). Discrete element simulations of direct shear tests with particle angularity effect. Granul. Matter.

[64] Zhou B, Wang J, Wang H (2018). Three-dimensional sphericity, roundness and fractal dimension of sand particles. Geotechnique.

